# Effect of Different Etching Times on Pit-and-Fissure Sealant Micro-Shear Bond Strength to the Enamel of Primary Teeth

**DOI:** 10.3390/children10030461

**Published:** 2023-02-26

**Authors:** Johnny Kharouba, Anna Annael Gonoratsky, Tamar Brosh, Mahmoud Masri, Rabea Iraqi, Sigalit Blumer

**Affiliations:** 1 Department of Pediatric Dentistry, The Maurice and Gabriela Goldschleger School of Dental Medicine, Tel Aviv University, Tel Aviv 69978, Israel; 2 Department of Oral Biology, The Maurice and Gabriela Goldschleger School of Dental Medicine, Tel Aviv University, Tel Aviv 69978, Israel; 3 Department of Oral Rehabilitation, The Maurice and Gabriela Goldschleger School of Dental Medicine, Tel Aviv University, Tel Aviv 69978, Israel

**Keywords:** enamel, etching, micro shear bond strength, pit-and-fissure sealants, primary teeth

## Abstract

Successful clinical use of pit-and-fissure sealants relies on the sufficient etching of the enamel, field isolation and sealant retention. The etching time changes the physical and mechanical surface properties of the etched tooth tissues; therefore, it impacts both etching depth and the bond strength of sealants to the enamel. We examined if reducing the recommended 15 s etching time of primary teeth enamel affects the micro-shear bond strength (µSBS) of pit-and-fissure sealants. The cusps of forty non-carious, extracted human primary molars were separately etched for 8, 15 or 30 s. Then, a pit-and-fissure sealant was placed and light-cured. The µSBS values were evaluated and compared among the three groups. The mean µSBS values ± standard deviations were 34.68 ± 16.93, 34.19 ± 17.35 and 36.56 ± 16.57 MPa in the cusps etched for 8, 15 and 30 s, respectively. No statistically significant differences in µSBS were observed among the three test groups. In this study, we showed for the first time that the recommended etching time of primary teeth enamel may be reduced from 15 to 8 s without compromising the µSBS of the sealant. Further evaluations in a clinical setting are warranted.

## 1. Introduction 

Pit-and-fissure sealants have been used for about 50 years to prevent and control carious lesions on primary and permanent teeth [1]. Sealants create a physical barrier between the oral environment and the occlusal tooth surface, thereby preventing the invasion of food and bacteria, and reducing the progression of occlusal caries lesions that have not been cavitated [1,2].

Three types of materials are used for sealing pits and fissures: resin-based sealants (RBS), glass ionomer cement-based sealants and polyacid modified resin sealants, with the first two as the most predominant [2]. RSBs are composed of urethane dimethacrylate or bisphenol A-glycidyl mathacrylate (bis-GMA) monomers and undergo polymerization by visible light at around 470 nm. Glass ionomer sealants are composed of fluoroaluminosilicate glass powder and an aqueous-based polyacrylic acid solution [1,2]. Some sealants comprise combinations of RSBs and glass ionomer cement-based sealants. For example, compomers are resin-based materials with additional fluoride-releasing properties, while resin-modified glass ionomers are glass ionomer sealants with additional resin components [1].

Primary teeth erupt between 6 months and 3 years of age and are replaced by permanent teeth by 12 years of age. Early childhood caries is defined as caries in children up to 72 months of age. The prevalence of early childhood caries in different countries around the world ranges from 23% to 90%, and in most of them it is higher than 50% [3]. Most caries in primary teeth is found in occlusal surfaces; therefore, fissure sealants can prevent the development of caries in these children. Moreover, the occurrence of caries in primary dentition, among other additional factors, is associated with the risk of developing carious lesions in permanent teeth of children and adolescents [4]. According to the guidelines of The American Academy of Pediatric Dentistry, the use of sealants is recommended over fluoride varnishes for sound occlusal surfaces and non-cavitated occlusal carious lesions in primary and permanent molars [5].

Sealants used on sound occlusal surfaces of non-cavitated pit-and-fissure carious lesions in primary or permanent molars demonstrated a reduction of 76% in the risk for developing new carious lesions after 2 years compared with control teeth that were not sealed. Moreover, children and adolescents with sealants had a lower caries incidence after ≥7 years of follow-up compared to those without sealants (29% vs. 74%) [6].

Sealants are less costly than extractions and restorations and can be applied quickly without local anesthesia; therefore, their use may prevent dental phobia, anxiety as well as dentist-avoidant behaviors [7].

Pit-and-fissure sealant materials have different characteristics. Their effectiveness is related to their retention rate, which may be affected by the chemical and physical properties of the sealant (e.g., viscosity, flow, wear/fracture resistance, bonding strength to the tooth structure) and their application technique (e.g., contamination control, tooth surface pretreatment, curing mode) [8]. The effectiveness of sealants has also been associated with the isolation of the working area because it helps maintain a dry area which is crucial to the success of the sealing procedure [9,10]. RSBs have good durability but they may shrink following polymerization. Such shrinkage may result in microleakage through which saliva and bacteria penetrate the occlusal barrier. Additionally, a stronger biofilm accumulation seems to occur on RSBs. Glass ionomer cement may fracture due to its reduced ability to withstand occlusal forces [1].

Placement techniques depend on the sealant type and its brand or manufacturer. Manufacturers’ instructions typically require cleaning and isolating the occlusal surface and recommend maintaining a dry environment during sealant placement and curing.

Before placing RSBs, occlusal surfaces must be etched [5]. Etching removes the smear layer and dissolves the tooth hydroxyapatite crystals, resulting in a relatively rough microporous surface that increases the surface area for micromechanical or chemical bonding [11,12]. After acid etching, the adhesive resin penetrates into the surface irregularities, thereby producing retentive tags [13]. Retention in resin-based pit-and-fissure sealants results from resin penetration into the porous enamel forming tags by capillary action, thereby leading to micromechanical interlocking between the resin and the enamel [14].

Etching time is defined as the duration of direct contact between the etchant and the dental hard tissues. The etching times changes the physical and mechanical surface properties of the etched tooth tissues; therefore, it impacts both the etching depth and bond strength of sealants to the enamel. A longer etching time increases surface roughness and mineral loss from the enamel surface, resulting in a loss of hardness [11]. The recommended etching time of permanent and primary enamel using Bis-GMA-based adhesive systems with 32–40% phosphoric acid is 15 s [15,16].

The disadvantages of phosphoric acid etching, particularly in children, who are the main users of sealants, increased patient anxiety during the treatment due to the time-consuming procedure and the acid’s bad taste [12]. Furthermore, adhesion using acid etching may fail due to plaque or pellicle retention, inadequate efficiency of etchant, tooth structures that are acid resistant, surface contamination by water or saliva and insufficient etching time [11].

The effects of etching time on the final bond and physical properties of the tooth surface area are not well understood. If the use of phosphoric acid etching is required for modifying enamel surface characteristics, rather than for significantly changing tooth substrate morphology, then it might be sufficient to shorten the phosphoric acid pre-etching times to less than 15 s [17]. Pediatric dentists mainly treat children of pre-school age and often seal primary teeth. In such patients and in children with special needs, it is difficult to maintain dryness during sealing, and therefore, shortening the etching time increases the chances of sealing success. Research is lacking in the field of primary teeth enamel etching and the relationship between the physical properties of pit-and-fissure sealants and short etching times of primary teeth enamel, probably because most of the studies to date have focused on permanent teeth [17,18].

Therefore, the purpose of this study was to examine for the first time whether reducing the current recommended etching time would maintain or reduce pit-and-fissure sealant micro-shear bond strength (µSBS) to the enamel of non-carious, extracted human primary molars.

## 2. Materials and Methods

### 2.1. Sample

To assess the sample size, we used G-power software under the following assumptions: type 1 error of 5%, desired minimum power of 80% and expected moderate effect size of the difference between time points (Eta squared = 0.20). Under these assumptions, the minimum sample size is 42 observations. However, of the 42 collected non-carious, extracted human primary molars, 2 broke, leaving 40 teeth in the experiment.

### 2.2. Tooth Preparation

Tooth preparations and all experiments were conducted by the same 2 researchers.

Forty non-carious, extracted human primary molars were randomly collected from several private dental clinics over a period of 3 months. The teeth were extracted due to periodontal reasons or physiological mobility. The teeth collected did not have caries or any developmental defect.

**Figure 1:**
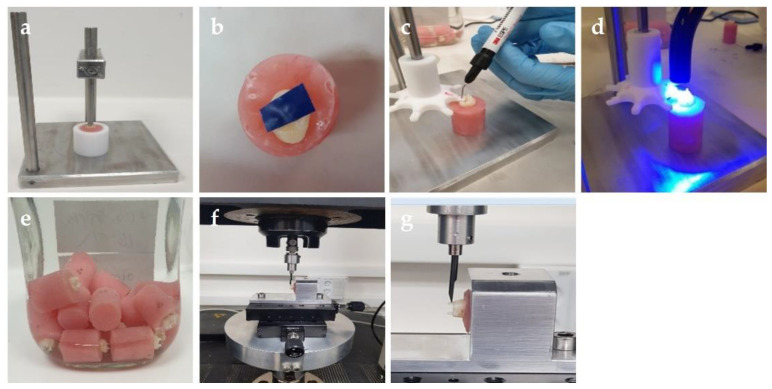
Experimental procedure. (**a**) The roots of the teeth were mounted on acrylic resin using a designated appliance and (**b**) cusps were protected using an isolating tape. (**c**) The pit-and-fissure sealant was applied as a cylinder using a designated device and (**d**) the teeth were cured with an LED light-curing unit. (**e**) The specimens were stored in a 0.1% thymol solution prior to the µSBS tests. (**f**) Each tooth was placed in the Instron machine. (**g**) The blade was placed at the interface between the enamel and the pit-and-fissure sealant, perpendicular to the occlusal surface of the tooth.

The teeth were rinsed under tap water flow and stored in a saline solution (0.9%) at room temperature until use. Each tooth was embedded in an acrylic resin (Coral-Fix, Henry Schein-Shvadent, Tel-Aviv, Israel) and mounted vertically using a designated device in a plastic ring mold (diameter = 20 mm, height = 25 mm) along the long axis of the tooth, in such a way that the crown of the tooth was completely exposed, and the rest of the tooth—from the cemento-enamel junction to the apex—was embedded in the acrylic resin. Blue sticky wax was placed on one side of the device, and on the other side of the tooth for grip and stabilization (Figure 1a).

Three tooth cusps of each molar were marked with the following etching times: (1) 8 s, (2) 15 s and (3) 30 s. These etching times were chosen to compare among the established etching times of 30 and 15 s and an etching time that is reduced by 50% to 8 s.

Each cusp was flattened by a flat head regular diamond bur (G1, wheel, code 818-030, Strauss & Co, Ra’anana, Israel) using a water spray turbine and was reduced by 1 mm of enamel to create an even, flattened occlusal surface. The flattened surface was confirmed to be enamel without dentin by using a probe that indicated the reduction of 1 mm, and by the lack of change of color of the tooth surface, as it is known that the dentin is more yellowish than the enamel. Flattening of all teeth was performed buccolingually by one researcher only to control for the different manual dexterities of different researchers. The operator controlled the air-turbine handpiece during cutting with a free-hand technique by putting both hands on a flat hard surface for motion stabilization: one hand holding the tooth and the other hand controlling the turbine. The approximate size of the obtained flat enamel on each cusp was a circle 3–2.5 mm in diameter.

To prevent leakage of the etching material, the cusps that were not yet prepared were covered with isolation tape (Figure 1b). Each cusp was etched for the chosen time using one drop of 37% phosphoric acid (Ultra-etch, Ultradent, South Jordan, UT, USA) (Figure 1c), with the other 2 cusps covered with an isolating tape. The surface was rinsed with water for 15 s and air dried for 15 s. Using the dedicated appliance, a light-cured, fluoride-releasing pit-and-fissure sealant (Clinpro™ 3M ESPE, Saint Paul, MN, USA) was placed in cylindrical shapes at a height of 3 mm and a diameter of 0.8 mm at the base of the flattened cusp (Figure 1c). The sealant was cured with a light-emitting diode (LED) emitting noncoherent blue light (ART-L5, Bonart Co. Ltd. New Taipei City, Taiwan), using a wavelength of 460–480 nanometers for 20 s (Figure 1d). In addition to the light cured intensity that was declared by the manufacturer, the lamps underwent power measurement by a radiometer (Maxima Curing Light Meter, Henry Schein, Melville, NY, USA). The fluoride was released from the sealant in a diffusion-limited process by exchange of hydroxide for the fluoride ion. The sealant has a color-change feature which aids the dental professional in the accuracy and amount of material placed during the sealant procedure: it is pink when applied to the tooth surface, and changes to an opaque off-white color when exposed to light. The teeth were stored for 7 days in a thymol 0.1% solution at room temperature prior to the µSBS test (Figure 1e). Thymol was used because of its well-known disinfectant and antioxidant properties [19].

### 2.3. µSBS Test

The µSBS test between the enamel and the pit-and-fissure sealant was performed using a loading machine equipped with 100 N load cell (Instron Model 4502, Instron Corp, Buckinghamshire, UK). Each specimen was mounted in a holder such that the metal knife blade was perpendicular to the treated occlusal surface of the tooth (Figure 1f). The metal knife blade was then moved vertically at the interface between the enamel and the pit-and-fissure sealant, at a cross head speed of 0.5 mm per minute, until the breakage point (Figure 1g).

The minimum load required to produce “bond fail” was determined from the first load drop on the load deflection plot. The data were collected automatically as a force in Newtons (N) when failure occurred, and were subsequently converted into megapascals (MPa = N/mm^2^) using the Material Testing program (Instron).

µSBS values were calculated using the equation:

F/π*r^2^, where: r = radius of cylinder in mm (0.4 mm), F= load at failure in N.

### 2.4. Statistical Analysis

Statistical analysis of the force and µSBS values was performed using one-way repeated measures analysis of variance (ANOVA) by SPSS (version 24.0, IBM Corporation, Armonk, NY, USA). The three etching times were compared within each tooth. The significance level was 0.05.

## 3. Results

**Figure 2:**
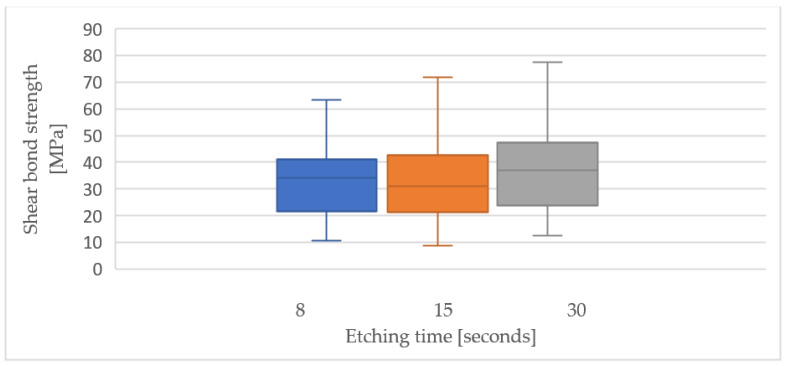
Box plot of µSBS value ranges and the median values of each group.

The three different etching times demonstrated similar values for the load needed for failure (Table 1) and for the µSBS applied (Table 2), as reported in Table 1 and Table 2. The group of samples that was etched for 30 s revealed the highest mean µSBS (36.56 ± 16.57 MPa). This result was higher than the mean µSBS at 8 and 15 s (34.68 ± 16.93 MPa and 34.19 ± 17.35 MPa, respectively, *p* value = 0.736) (Table 2 and Figure 2).

Mauchly’s test of sphericity was not significant, indicating that sphericity could be assumed when interpreting within-subject effects (χ^2^(2) = 1.41, *p* = 0.494).

Within-subject tests (Table 3) revealed insignificant difference among the three groups of etching times: (F (3.78) = 0.307, *p* = 0.736). Thus, there was no statistical difference between the shear bond values amongst the three different etching times: 8, 15 and 30 s.

**Figure 3:**
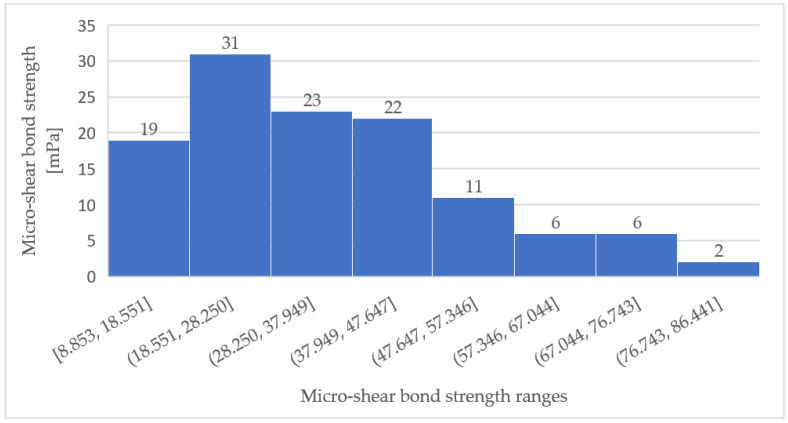
Micro shear bond strength distribution of all 120 specimens.

In the absence of statistically significant differences in µSBS among the three experimental groups, we examined the distribution in µSBS values among all 120 samples (Figure 2). The highest number of samples, for µSBS values ranging from 18.55 to 28.25 MPa, had (*n* = 31) (Figure 3).

## 4. Discussion

Bond strength is a reliable parameter for quantifying sealants’ adhesivity to enamel substrates and is considered an essential indicator of the clinical situation [17,20]. Although adhesive bond strength can also be measured by micro-tensile loads, according to the Academy of Dental Materials, shear bond strength tests are valid for measuring the adhesion on enamel [21], and this method is well established [22,23]. Shear bond strength testing with bonded cross-sectional areas of 3 mm^2^ or less is considered μSBS [22]. A significant advantage over micro-tensile strength methods is that the μSBS specimen is pre-stressed prior to testing only by mold removal [22]. In the present study, the approximate size of the obtained flat enamel on each cusp was a circle 3–2.5 mm in diameter, which allowed to create the cylindrical form of 0.8 mm diameter at the base of the cylinder. Hence, µSBS was tested [24,25].

Primary teeth have lower mineral content and thinner dental tissues than permanent teeth [26]. The tubule density of primary dentin is higher with a larger diameter in peritubular and intertubular dentin [27]. The enamel of primary teeth has an outer prismless layer. It was previously thought that the mineral content of primary tooth enamel makes adhesion to it less reliable than to permanent tooth enamel [28], but a systematic review of studies that examined adhesive bond strength in primary and permanent teeth concluded that permanent tooth dentin has higher bond strength than primary tooth dentin, while primary and permanent tooth enamel have similar bond strengths [29]. Our findings show that the µSBS values were not statistically significantly different among three tested etching times (8, 15, 30 s), ranging from 34.19 to 36.56 MPa. The µSBS of 63% of samples (76/120) ranged from 18.55 to 47.65 MPa. These values enable a good retention rate. The similar µSBS values obtained for the three test groups may indicate the similar conditions that were created in the enamel. These values were higher than those reported by Pushpalatha et al. [30] who used the Clinpro sealant on primary molars after etching for 20 s to achieve µSBS of 27.6 MPa.

The application of conventional resin-based pit-and-fissure sealants is a very sensitive and time-consuming technique, which requires maintaining a dry field in order to create an effective bond [12]. The current recommended 15 s acid etching time allows the enamel to be etched while the underlying dentin is demineralized 5–8 µm, making its infiltration easier [31]. Due to this clinical advantage, an etching time of 15 or 30 s has become the prevailing method of acid etching in recent years [31]. The etching times chosen in the current study were intended to examine the maximum and minimum etching times (8 and 30 s, respectively) around 15 s of the currently recommended etching time, for obtaining similar results of µSBS. The reduction in the etching time to half of the current recommended time while maintaining similar µSBS values is expected to decrease surface degradation and to be more biocompatible with the enamel and the dentin. Moreover, it will allow for reducing chair time and providing faster treatment, thereby increasing the chance of keeping a dry work field as well as patient cooperation and reducing the resistance of young patients during treatment. It is important to note that if a child wets the tooth with saliva, then the whole etching process must start again. If the child does not cooperate with the dentist, the clinician may not repeat the etching process despite contamination by saliva, and this will affect bonding and sealant retention [32]. In addition, the taste of the acid can prevent the child from continuing the treatment, which can increase their anxiety about the treatment and further treatments. Our findings are also significant for other situations in which dentists face a challenge to provide dental care, for example, when treating children with special needs.

Others have also attempted to shorten the etching time in primary teeth. Nordenvall et al. [33] have found that resin tags formed in primary enamel that was etched for 60 and 15 s were not statistically significantly different, with areas of atypically rounded irregularities, which exhibited a granulated structure. Primary teeth demonstrated the highest surface irregularity scores following etching for 15 s. According to Pushpalatha et al. [30], the µSBS of Clinpro^TM^ sealant to primary tooth enamel was slightly higher than µSBS to the enamel of permanent teeth, but in general, retention and sealant success were equivalent in both tooth types [34].

The lack of statistically significant differences in µSBSs obtained by the different etching times in our study may be explained by the similar etching depths, which resulted in similar resin tag lengths. Sound enamel etching causes microscopic level changes, which form three zones. The first zone is a reactive surface, approximately 10 µm in depth, which is formed by the removal of a narrow zone of enamel as well as plaque, surface, subsurface organic pellicles and inert mineral crystals. The second zone formed by etching is a qualitative porous zone, 20 µm in depth. The third is a quantitative porous zone, 20 µm in depth. The pit-and-fissure sealant applied to the etched enamel penetrates the microporosities and forms resin tags [33,35]. Although different tag lengths may form in this process, it seems that they do not affect the µSBS of the tested etching times. Further research should examine the impact of shortened enamel etching times on resin tag length.

The high values of µSBS attained in the study may be explained by the specific failure type that was created. We can assume that cohesive failure inside the material was predominant among the results, resulting in higher µSBS in comparison to previous studies.

As only a minimal amount of enamel should be dissolved from the tooth surface, only the minimal etching time that is appropriate for achieving optimum bonding should be used. A shorter etching time may be clinically significant if it allows for long-term bond strength. Shortening the etching time from 15 to 8 s may help in patient management, particularly pediatric patients, and the protocol for such treatment should be acceptable if it reduces chair time without compromising the result. However, to reach conclusive evidence, additional mechanical and physical properties, such as microhardness, marginal leakage, free surface energy and length of the resin tags should be evaluated, in addition to µSBS.

The longevity of the sealant placed inside the tooth fissures depends on the retention of the material and its ability to act as a barrier between the oral environment and the tooth. Sealant retention may be affected by isolation, use of bonding agents, enameloplasty and maintenance [36]. Saliva contamination reduces the bond strength significantly. Coelho et al. showed that sealant adhesion mediated by phosphoric acid conditioning was highly unsatisfactory and unpredictable when the enamel was contaminated by saliva or water and concluded that the application of fissure sealants according to the classical technique is indicated only in clinical conditions that ensure excellent isolation and moisture control of the surface [32]. The patient’s behavior and compliance also play a significant role in sealant retention studies [34]. Our findings suggest that etching for 8 s will provide the same sealant retention as etching the enamel for 15 or 30 s, but further clinical research is warranted.

As the present investigation was an in vitro study, it has somewhat limited external validity. On the one hand, the bond strength of the sealant at 8 s of etching was similar to that at 15 and 30 s of etching, and it is sufficient for the optimal retention of the sealant. On the other hand, the exact conditions of the oral cavity cannot be mimicked. The chewing forces can also affect the retention of the sealant, which is difficult to duplicate in an in vitro study. Additionally, the laboratory has optimal conditions for maintaining tooth dryness, and the parameter of child cooperation is missing.

The structure of the “enamel surface” is different from that of the underlying enamel structure; therefore, in clinical practice, phosphoric acid etching is performed on “uncut” enamel prior to applying the sealant. This could not be carried out in the current study because of the cylindrical shape of the sealant specimen. To obtain a uniform contact area, all samples were attached to a flat surface. The surface roughness of the cut enamel could have influenced the sealant’s µSBS; however, had we tried to imitate the method for sealing the pits and fissures as it is actually carried out in the clinic (by using carbide round burs or by not using burs at all), we would not have been able to standardize all forty teeth, nor to examine them in the Instron machine. Bond strength is influenced by the hardness of the sealant if there were many cohesive failures in material; however, the mode of fracture was not analyzed in this study. Bond durability was evaluated by water immersion; therefore, the outcome may be different in an oral environment. Additionally, the low viscosity of the tested sealant made it difficult to control the cylindrical shape of the sealant. Another issue was the removal of just enough enamel without reaching the dentin layer. As this depends on the researcher’s manual dexterity, it was standardized by conducting all experiments by the same two researchers. In the clinic, etching is followed by washing the tooth and drying it prior to applying the pit-and-fissure sealant. The actual dryness and the isolation conditions differ from one patient to another, and depend on the amount of saliva, the clinician’s ability to control moisture, the use of a rubber dam and the patient’s age and cooperation with the treatment. This variable was not checked in the current study. Another limitation is that the specimens were kept at room temperature but should have been kept at the same temperature of the oral cavity (37 °C) to simulate the oral environment. Sealant aging before the bond strength tests is an additional limitation. In the current study, µSBS tests were performed after storing the prepared teeth in a thymol solution for a week. In the clinic, sealants’ longevity can last up to years before they are deboned. Therefore, further research should be carried out to support the conclusions of the study.

## 5. Conclusions

This study showed that the etching of primary teeth enamel for 8 s results in similar pit-and-fissure sealant µSBS values compared to etching for 15 or 30 s. Therefore, within the limits of this in vitro study, a reduction in the recommended etching time of primary teeth enamel from 15 to 8 s is possible without compromising the µSBS of the pit-and-fissure sealant. Further research should examine the use of shorter etching times on enamel-etched surfaces and evaluate marginal leakage, failure site and type and compare etching patterns using scanning electron microscopy or other imaging tools. Clinically, the reliability of a shortened etching time in the oral environment should be demonstrated.

## Figures and Tables

**Table 1:** Load values needed for the Clinpro^TM^ pit-and-fissure sealant bond failure after different etching times in non-carious, extracted human primary molars.

Etching Time	Number of Teeth	Sum of Load	Mean Load ± SD (N)	Variance
8 s	40	697	17.43 ± 2.6	7.24 × 10^−2^
15 s	40	687	17.19 ± 2.7	7.6 × 10^−2^
30 s	40	735	18.38 ± 2.5	6.94 × 10^−2^

SD = standard deviation.

**Table 2:** µSBS values between the enamel of non-carious, extracted human primary molars and the ClinproTM pit-and-fissure sealant after different etching times.

Etching Time	Number of Teeth	Sum of µSBS (MPa)	Mean µSBS ± SD (MPa)	Variance	*p* Value
8 s	40	1387.19	34.68 ±16.93	286.54	0.736
15 s	40	1367.54	34.19 ±17.35	301.16	
30 s	40	1462.56	36.56 ±16.57	274.71	

SD = standard deviation.

**Table 3:** Assessment of within-subject effects of shear bond values by etching time.

Source		Sum of Squares	Degree of Freedom	Mean Square	F-Value	*p*-Value
Etching time	Sphericity Assumed	125.778	2	62.889	0.307	0.736
Error	Sphericity Assumed	15,961.499	78	204.635		

## Data Availability

The data that support the findings of this study are available from the corresponding author, upon reasonable request.
